# Insights into the mechanism of a novel shockwave-assisted needle-free drug delivery device driven by in situ-generated oxyhydrogen mixture which provides efficient protection against mycobacterial infections

**DOI:** 10.1186/s13036-017-0088-x

**Published:** 2017-12-12

**Authors:** Janardhanraj Subburaj, Akshay Datey, Jagadeesh Gopalan, Dipshikha Chakravortty

**Affiliations:** 1 Department of Aerospace Engineering, Indian Institute of Science, Bangalore, India; 2 Department of Microbiology and Cell Biology, Indian Institute of Science, Bangalore, India; 3 Centre for Biosystems Science and Engineering, Indian Institute of Science, Bangalore, India

**Keywords:** Shockwaves, Oxyhydrogen, *Mycobacterium bovis* BCG, *Mycobacterium tuberculosis*, Vaccination, Immune response

## Abstract

**Background:**

Needle-free, painless and localized drug delivery has been a coveted technology in the area of biomedical research. We present an innovative way of trans-dermal vaccine delivery using a miniature detonation-driven shock tube device. This device utilizes~2.5 bar of in situ generated oxyhydrogen mixture to produce a strong shockwave that accelerates liquid jets to velocities of about 94 m/s.

**Method:**

Oxyhydrogen driven shock tube was optimized for efficiently delivering vaccines in the intradermal region in vivo. Efficiency of vaccination was evaluated by pathogen challenge and host immune response. Expression levels of molecular markers were checked by qRT-PCR.

**Results:**

High efficiency vaccination was achieved using the device. Post pathogen challenge with *Mycobacterium tuberculosis*, 100% survival was observed in vaccinated animals. Immune response to vaccination was significantly higher in the animals vaccinated using the device as compared to conventional route of vaccination.

**Conclusion:**

A novel device was developed and optimized for intra dermal vaccine delivery in murine model. Conventional as well in-house developed vaccine strains were used to test the system. It was found that the vaccine delivery and immune response was at par with the conventional routes of vaccination. Thus, the device reported can be used for delivering live attenuated vaccines in the future.

**Electronic supplementary material:**

The online version of this article (10.1186/s13036-017-0088-x) contains supplementary material, which is available to authorized users.

## Background

The most commonly used needle and syringe method for drug administration has come under scrutiny in the recent decades because of factors like needle contamination, requirement for safe disposal of used needles, waste accumulation, accidental needle-stick, pain during usage and needle phobia [1]. Therefore, many new alternative non-invasive means of drug delivery have been developed, which mainly use oral, pulmonary, nasal, buccal or transdermal routes of administration [1–3]. Among these, the drug transport through human skin proves to be more advantageous compared to the other routes due to the ease of administration, immuno-surveillance functions and easy accessibility [2, 4]. Liquid-jet injectors, powder immunization and microneedles are some of the budding technologies for transdermal delivery of drugs [1–6]. Many methods to improve the efficiency of transdermal therapeutic systems by enhancing the driving force to increase the rate of drug transport have also been suggested [6, 7]. However, these techniques face major challenges due to the selectively permeable nature of the human skin and its ability to restrict molecular transport.

Shockwaves are non-linear waves, propagating at speeds greater than the speed of sound, with a unique characteristic of instantaneously increasing the pressure, temperature and density of the medium through which they propagate. For over a half a century, the phenomena of shockwaves have been synonymous with aerospace research [8, 9]. The emerging paradigms of present day shock wave research have opened up new horizons for interdisciplinary applications [10]. Shockwaves have been extensively used for various medical procedures like extracorporeal lithotripsy [11], treatment of avascular necrosis [12], accelerated bone fracture healing [13], angiogenesis [14] and tendinitis [15]. The use of shockwaves as a driving force for transdermal drug delivery has proved to be effective because of their ability to accelerate the drug particles to high velocities so that they can penetrate the skin [16–21].Shockwaves have also been demonstrated to generate high velocity projectiles for delivering nucleic acids into living cells [22–25]. However, the use of either compressed air bottles [16–18], ignition of detonable mixtures [20–25] or operation of bulky and expensive instruments [19] to generate shockwaves, make these techniques undesirable. Also, many of the techniques result in the accumulation of waste and production of harmful by-products during the detonation of mixtures.

In the present work, we have developed a shockwave-assisted vaccine delivery device that can generate high velocity jets through the detonation of in situ generated oxyhydrogen mixture (stoichiometric mixture of hydrogen and oxygen gases in the ratio 2:1). BCG and *Salmonella* vaccine strains (DV-STM-07) [26] have been successfully injected using this device in the mice model. The device has been optimized to obtain penetration depths of ~100 μm in the skin. The molecular mechanism of the immune response to shockwave mediated vaccine delivery has been worked out. The ability of the proposed device to produce shockwaves of required strength in a safe, clean and reproducible manner opens up new opportunities for shockwave-assisted biomedical research. This device demonstrates the potential for localized drug delivery using shockwaves.

## Methods

### Operation of the oxyhydrogen-driven drug delivery device

The biological sample was placed in the sterile cavity and clamped at the end of the shock tube. Tracing paper was used between the driver and driven section of the shock tube. The electrolysis process in the oxyhydrogen generator was initiated when the power supply from the DC source was switched on. The oxyhydrogen generator produced stoichiometric ratio of hydrogen and oxygen gases at around 8 ml/s. As soon as the fill pressure in the driver section of the shock tube reached2.5 bar, the power supply was switched off. By evacuating the pressure between the oxyhydrogen generator and the miniature shock tube, it was ensured that the detonation front does not travel into the oxyhydrogen generator when the shock tube is operated. The miniature shock tube was operated by igniting the mixture using a spark plug. For the subsequent run, the ruptured paper diaphragm was replaced with new ones. The biological sample was replaced with a new sample.

### Pressure measurements in the setup

The pressure in the shock tube was measured using a piezoelectric pressure sensor (PCB Piezotronics, USA). This sensor was flushed mounted to the wall of the shock tube. To measure the head-on pressures experienced by the liquid in the cavity, the sensor was mounted on the base of the cavity. A polyvinyldifluoride (PVDF)-coated needle hydrophone (Dr. Müller Instruments, Germany) was used to measure the pressure experienced by the bacterial culture in the cavity. The needle gauge was placed in the cavity so that the tip of the sensor is in contact with the bacterial culture. All the pressure signals obtained from the sensors were recorded in an oscilloscope (Yokogawa Electric, Japan).

### High-speed visualization of the blast wave evolution and liquid jet

A conventional Z-type Schlieren visualization technique that uses a high-speed camera (Phantom V310, Vision Research, USA) and an LED light source were used to obtain time resolved high speed images. The Schlieren system was triggered using the pressure sensor at the end of the driven section.

### Institutional ethical clearance and permission

All the experiments were carried out in accordance with the institutional ethical guidelines. Ethical clearance for the project was obtained from the Institutional Animal Ethics Committee (IAEC; Reg. no. 48/1999/CPCSEA, Chairman: Prof. D N Rao). Permission no: CAF/Ethics/380/2014.

### Effect of shock waves on the viability of bacteria

*Salmonella* Typhimurium and *Mycobacterium bovis* BCG were the model microorganisms used to study the effect of shock waves on bacterial viability. Overnight cultures were pelleted and suspended in sterile phosphate buffered saline (PBS). The re-suspended culture was exposed to shockwaves at different pressures. The cultures were immediately aspirated, serially diluted and plated on SS (Salmonella-Shigella) agar or Middlebrook 7H10 agar. Equal volume of unexposed culture was used as a control. CFU were counted and was expressed as percent viability.

### Depth of penetration measurements

To mimic the physical parameters corresponding to the animal skin, 20% Polyacrylamide gel was used to visualize the injection. Carboxylate-modified polystyrene fluorescent yellow-green Latex beads (L4655, Sigma-Aldrich) (4.6 × 10^9^ particles per ml) were injected into the polyacrylamide gel using the device. The gel was immediately visualized using a Carl Zeiss 710 confocal microscope to visualize and quantify the depth of penetration. Above mentioned latex beads were injected in vivo on the dorsal side of the animal. The skin was removed and mounted on a glass slide and immediately visualized using confocal microscope. Images were obtained in *xyz* scanning mode and captured every 2 μm from the skin surface until no appreciable fluorescence could be detected.

### Immunization of the mice

BALB/c mice were bred and housed at the Central Animal facility, Indian Institute of Science. The mice used for the experiments were 6–8 weeks old. All procedures with animals were carried out in accordance with the institution-approved rules. The abdominal region of the animal was shaved using hair removal cream. The skin was wiped with 70% ethanol before the vaccine delivery. DV-STM-07 and *M. bovis* BCG were used as vaccine strains. The mice were held in close proximity to the device. The device was operated at an optimum fill pressure of 2.5 bar to ensure the injection of the vaccine. Mice (*n* = 5 per group) were immunized using approximately 10^3^ bacteria/mouse. One group of mice was administered PBS which served as a control. Mice in the positive control group for DV-STM-07 vaccination received 10^7^ CFU/mouse via the oral route. 1-week post immunization all the mice were challenged orally with 10^7^ CFU/ml and 10^8^ CFU/ml of virulent *Salmonella* Typhimurium for estimating the organ burden and for survival assay respectively. The mice were monitored twice daily for the morbidity and mortality.

### BCG vaccination

BCG vaccine was administered to animals using the device (approximately 10^3^ CFU/animal) as described above. A booster dose was given on day 7 post primary dose of the vaccine. Positive control group was administered the vaccine via intradermal route.

### Estimation of organ burden in BCG vaccination

Day 14 post vaccination, animals were challenged with *Mycobacterium tuberculosis H37Ra* (10^7^ CFU/mouse) via the nasal route using agarose bead slurry. Day 7 post infection, the animals were sacrificed using cervical dislocation method and the lungs were aseptically dissected, homogenized and plated on 7H10 agar. CFU were counted and were represented as CFU/g organ weight.

### Estimation of organ burden in DV-STM-07 vaccination

Five days’ post oral challenge the mice were sacrificed by the cervical dislocation method^32^. The liver, spleen and mesenteric lymph nodes (MLN) were aseptically isolated, weighed and homogenized in sterile PBS. The homogenate was serially diluted and plated on SS agar to estimate the bacterial load in various organs. CFU were counted and represented as CFU/g wt. of the organ.

### Estimation of serum immunoglobulin-G (IgG)

Blood was collected from the mice by retro-orbital ocular bleeding (33). Serum was isolated by centrifugation and stored at −80 °C until use. The serum samples were diluted in PBS supplemented with 3% bovine serum albumin (BSA) prior to use in enzyme-linked immune sorbent assay (ELISA). Serum Immunoglobulin-G (IgG) titer specific to Salmonella lipopolysaccharide (LPS) and BCG was estimated using ELISA. LPS (250 ng/100 μl/well) in 0.02% trichloroacetic acid or BCG bacteria (10^3^ CFU/100 μl/well) were coated in a 96 well plate and incubated at 4 °C for 12 h. The unbound sites on the plate were blocked with 3% BSA for 1 h at room temperature. 100 μl of diluted serum (1:10) was added and incubated for 1 h at 37 °C and was washed. HRP conjugated anti IgG mouse (1:5000 dilutions) was added and incubated for 1 h at 37 °C. The wells were washed with PBS with Tween 20 thrice. 100 μl of tetramethylbenzidine solution was added and incubated for 15 min. 50 μl of 1 N H_2_SO_4_ was added to stop the reaction and the absorbance was recorded at 540 nm. The highest dilution at which the absorbance of the sample exceeds the background absorbance by 2 standard deviations was taken as end point titers of the sera.

### Estimation of CD8^+^ T cells

Splenic T cells were isolated from unvaccinated and vaccinated mice as follows: Freshly harvested splenic tissue was physically disintegrated using roughened glass slides in tissue culture medium maintained at 4 °C. Single cell suspension was made by passing the cell suspension though a filter. RBC lysis was done using RBC lysis buffer. After lysis, cells were allowed to adhere to culture dishes for 1 h. Supernatant containing T cells was collected. Cells were briefly washed with sterile PBS and were fixed using 3% paraformaldehyde for 15 min and were stored at 4 °C until further use. Cells were stained with PE tagged antiCD8 antibody (MiltenyiBiotec) and were analyzed by flow cytometry. Numbers of CD8+ cells were compared between the unvaccinated and the vaccinated cohort.

### Quantitative real time PCR

Tissue was aseptically isolated from the animal and was immediately resuspended in Trizol (Invitrogen) and stored in −80 °C till further use. RNA was isolated and converted to cDNA using reverse transcriptase. Real time PCR was set up using SyBr green kit from Thermo Scientific and was run on Applied Biosystems VIIA. The fold expression was normalized to ß-actin.

### ELISA for serum cytokines

Blood was collected through the retro-orbital route. Serum was isolated by centrifugation and was stored at −80 °C until use. BD Opt ELISA kit was used for determining the levels of cytokines in the serum.

### Statistical analysis

The data sets were statistically analyzed by applying Student’s t-test and Mann-Whitney U test using Graph Pad prism 5 software. A *p*-value <0.05 was considered as significant. The mortality and survival data were analyzed by survival curve analysis. All the experiments were repeated at least thrice to validate the results.

## Results

### Design of the oxyhydrogen detonation-driven drug delivery device

**Fig. 1:**
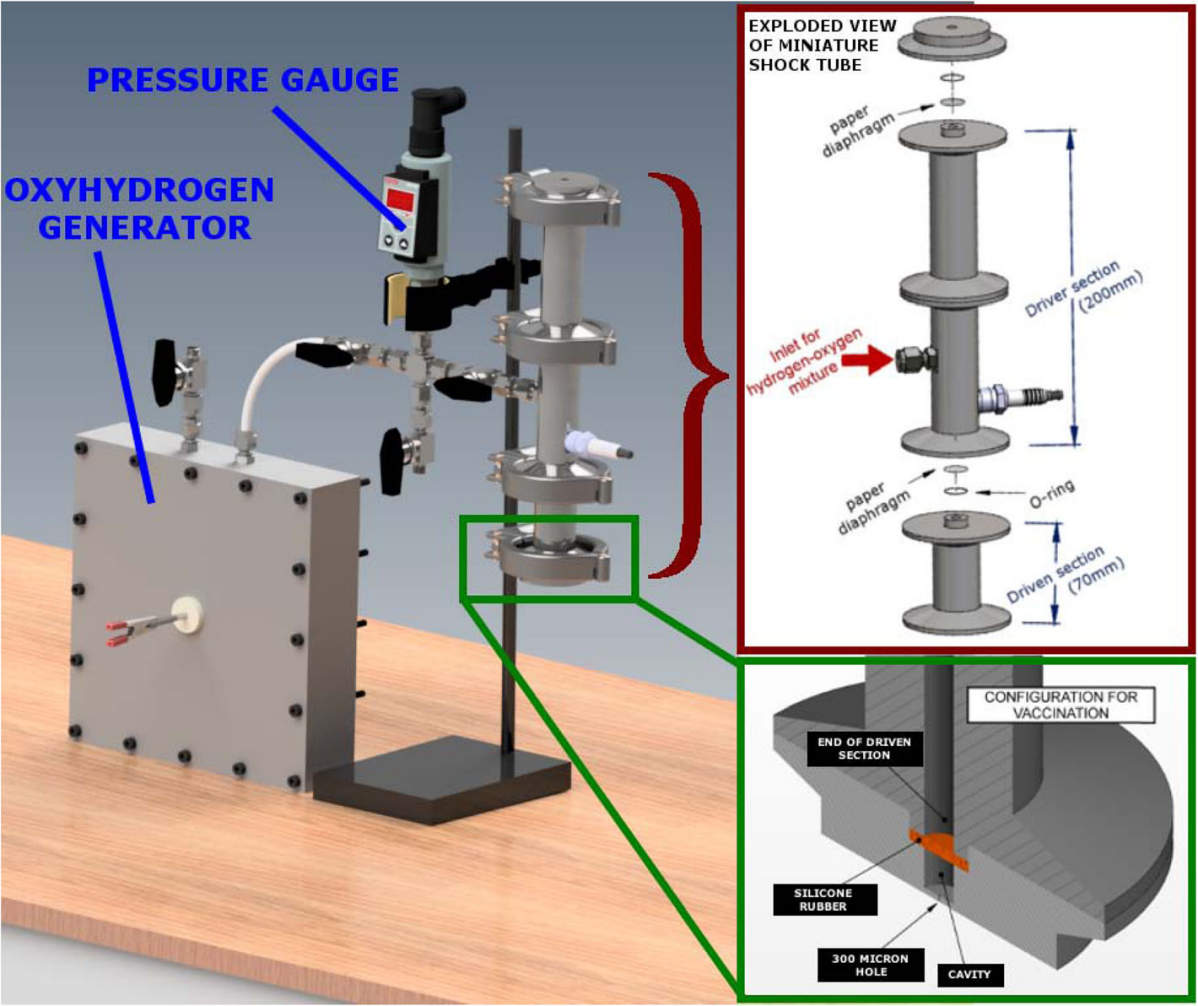
Schematic of the oxyhydrogen detonation-driven drug delivery device. Schematic of the entire experimental setup showing the various subcomponents. Cross-sectional view of the end of the miniature shock tube assembly showing the flexible silicone rubber and the 300-μm hole in the cavity

The oxyhydrogen detonation-driven drug delivery device comprises of two main components namely oxyhydrogen generator and miniature shock tube assembly. While the design and working of the oxyhydrogen generator used for the present work has already been described [27], some changes have been made in the miniature shock tube assembly to facilitate vaccine delivery experiments (Fig. 1). Through alkaline electrolysis, the oxyhydrogen generator produces about 2.5 bar of oxyhydrogen mixture during each operation of the device. The miniature shock tube of internal diameter 6 mm comprises of two sections – a driver section of length 200 mm and driven section of length 70 mm (Fig. 1). Tracing paper (95 GSM) is used as diaphragm to separate the driver section and driven section of the shock tube. To facilitate quicker and easier changing of diaphragm after each experiment as well as filling the drug in the cavity, a tri-clover clamp is used between the different sections of the shock tube. The driver section of the shock tube is filled with 2.5 bar of oxyhydrogen mixture and the mixture is detonated. This ruptures the diaphragm and a strong shockwave is created in the driven section of the shock tube. The working of the miniature shock tube is explained in a schematic diagram (see Additional file 1: Figure S1). The vaccine is accommodated in a stainless steel sterile cavity of diameter 6 mm and depth 5 mm (Fig. 1). The dimensions of the cavity is fixed based on a study performed earlier [21]. The bottom of the cavity has a 300 μm diameter hole to allow vaccine to be ejected in the form of a jet on shockwave impact. The drug is held back initially in the cavity by surface tension. The direct impact of the shockwave followed by the products of detonation on the drug sample leads to issues of contamination. To avoid this, a suitable barrier is chosen for good energy transfer and to prevent the detonation products from impacting the drug. Using a brass foil is a viable option [20] but requires replacement after every experiment and also it absorbs most of the incident shockwave energy before transmitting it to the biological sample. Therefore, in the present workman silicone rubber membrane has been used between the shock tube and the drug. Silicone rubber has biocompatible properties and very good tensile strength [28].These properties make it ideal for the present work as it need not be replaced frequently and the energy transfer is better as compared to using the brass foil.

### Performance of the oxyhydrogen detonation-driven drug delivery device

**Fig. 2:**
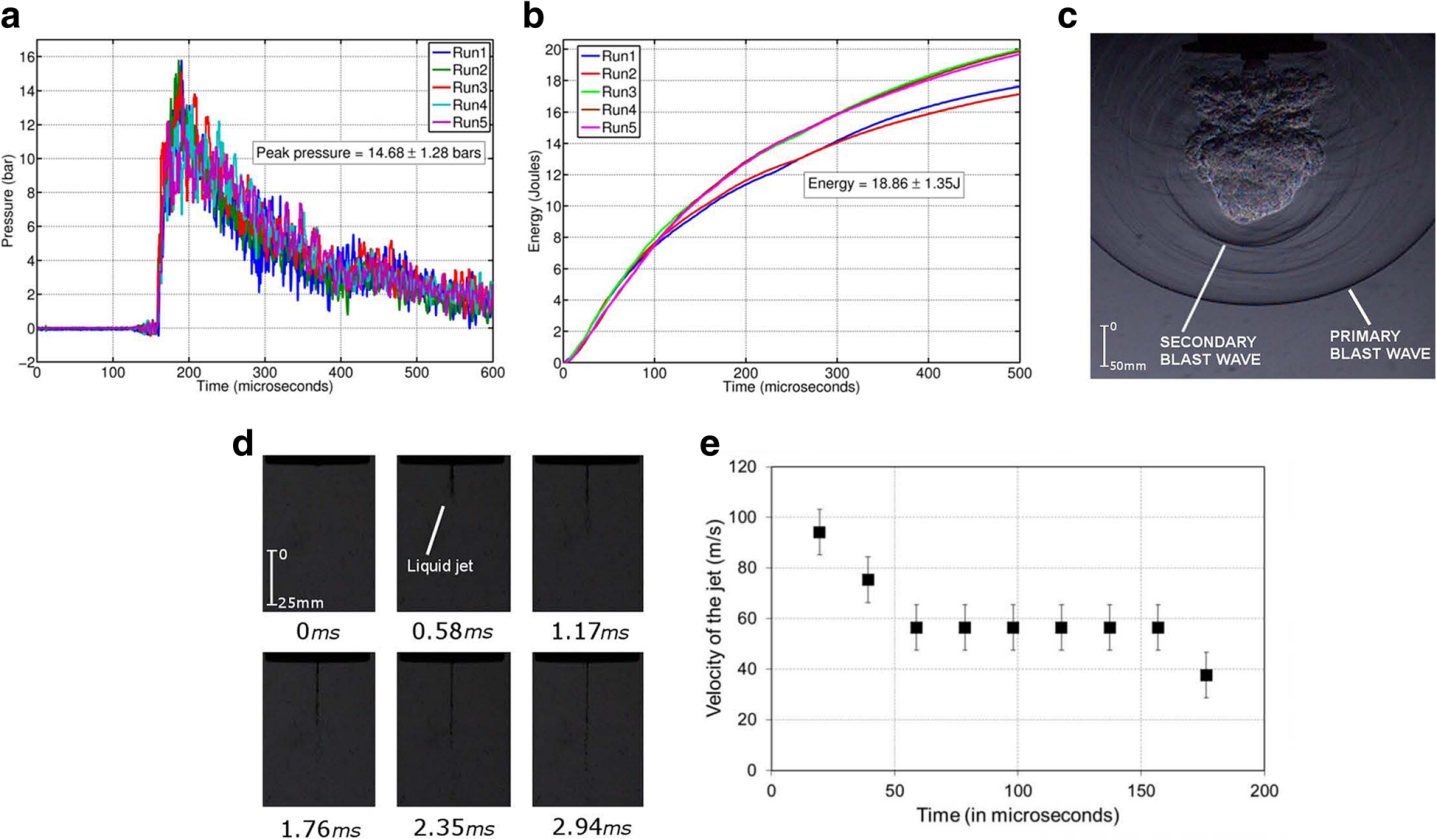
Measurements made in the oxyhydrogen detonation-driven miniature shock tube. **a** Typical pressure signals for five different runs of the shock tube operated at an oxyhydrogen fill pressure of 2.5 bar. The pressure sensor is flush-mounted to the inside wall of the driven section at a distance of 12 mm from the end. The driven section is left open to the atmosphere. **b** The corresponding energies in the shock wave for the five runs. **c** Time resolved high speed Schlieren images of the blast wave emanating from the open end of the shock tube. The images are acquired at a frame rate of 40,000 fps. **d** Time-resolved high speed shadowgraph images of the jet of drug ejected through the 300-μm hole. The images are acquired at a frame rate of 51,000 fps. **e** A velocity-time plot of the tip of the jet as measured from the high-speed shadowgraphy

The initial fill pressure of the oxyhydrogen mixture in the shock tube determines the strength of the shockwave produced which, in turn, determines the amount of energy transferred to the vaccine. In order to fix the initial fill pressure of the oxyhydrogen mixture for the experiments in the present study, we delved into the working of a previously reported shockwave-assisted drug delivery device that uses a Nonel tube to generate shockwaves. After an elapsed time of 50 μs, the blast wave emanating from the open end of the Nonel tube carries an energy of about 1.25 ± 0.94 J [29]. The energy of the blast waves produced using the present device was targeted to be around this value. The initial experiments using the miniature shock tube assembly was carried without the cavity, silicone rubber and the vaccine. The driven section was left open to the atmosphere to analyze the blast waves coming out of the open end of the shock tube. The miniature shock tube was operated with an initial oxyhydrogen fill pressure of 2.5 bar. Shockwaves with a peak pressure of 14.68 ± 1.28 bar were produced with good reproducibility at the end of the shock tube (Fig. 2a). The energy of the shockwave was estimated from the pressure-plot of the shockwave based on a procedure that has been reported elsewhere [30]. The energy of the blast waves emanating from the open end was estimated to be 18.86 ± 1.35 J after 500 μs (Fig. 2b). After 50 μs, the energy in the blast wave was about 4 J which is almost three times the targeted value. High speed schlieren photography [31] of the open end of the shock tube revealed the symmetrical nature of the blast wave about the axis of the shock tube(Fig. 2c and Additional file 2: video 1). The stronger color gradients indicate the higher strength of the primary and secondary blast waves emanating as a result of the oxyhydrogen detonation. Also, the cloud of detonation products behind the secondary blast waves could be observed in the photographs.

### Characterization of liquid jet delivery in artificial and animal skin

**Fig. 3:**
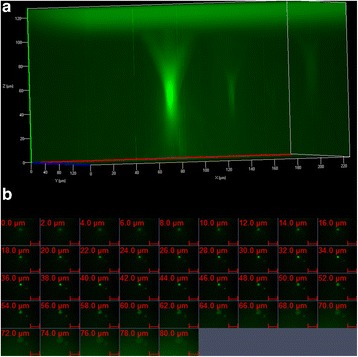
**a** Confocal images showing the penetration of fluorescent yellow-green latex beads delivered to 20% acrylamide gels using the device. **b** Confocal images (xyz scanning) showing the penetration of fluorescent beads delivered to the abdominal region of mouse skin using the device

High-speed images of the liquid jet were acquired using conventional Schlieren technique for oxyhydrogen initial fill pressure of 2.5 bar (Fig. 2d and Additional file 3: video 2). The velocity calculation from the images gave a value of 94 ± 9 ms^−1^ (Fig. 2e). The targeted values for velocity of jet and depth of penetration are ~100 ms^−1^and 100–150 μm respectively [20]. The aim of needleless vaccination is to deliver the biological sample to the stratum corneum layer of the skin which is known to be devoid of nerve endings responsible for the sensation of pain [5]. This portion of the skin is populated by special immune cells called Langerhans cells. These are the “professional” antigen presenting cells operating in the dermal region [5]. To check the depth of penetration of the liquid jet, initially 20% Polyacrylamide gel target (a known artificial skin model [32, 33]) was used. This gel has similar mechanical properties as compare to the human skin. The Young’s modulus of the human skin ranges from 0.02 to 57 MPa and that of the polyacrylamide gel used is <60 MPa [32]. Green fluorescent beads (1 μm diameter) were injected into the gel using the shock tube. The depth of penetration was evaluated using confocal microscopy, which was found out to be approximately 120 μm (Fig. 3a). This depth in the skin is the most suitable for painless injections. To validate the same in vivo, the beads were injected in the mice, the skin was dissected immediately and was analyzed using confocal microscope. The maximum depth of penetration of the beads was found out to be 80 μm which was reasonably sufficient to deliver the vaccine to the resident Langerhans’s cells for generating an immune response (Fig. 3b).

### Efficiency of BCG vaccination delivery using the device

**Fig. 4:**
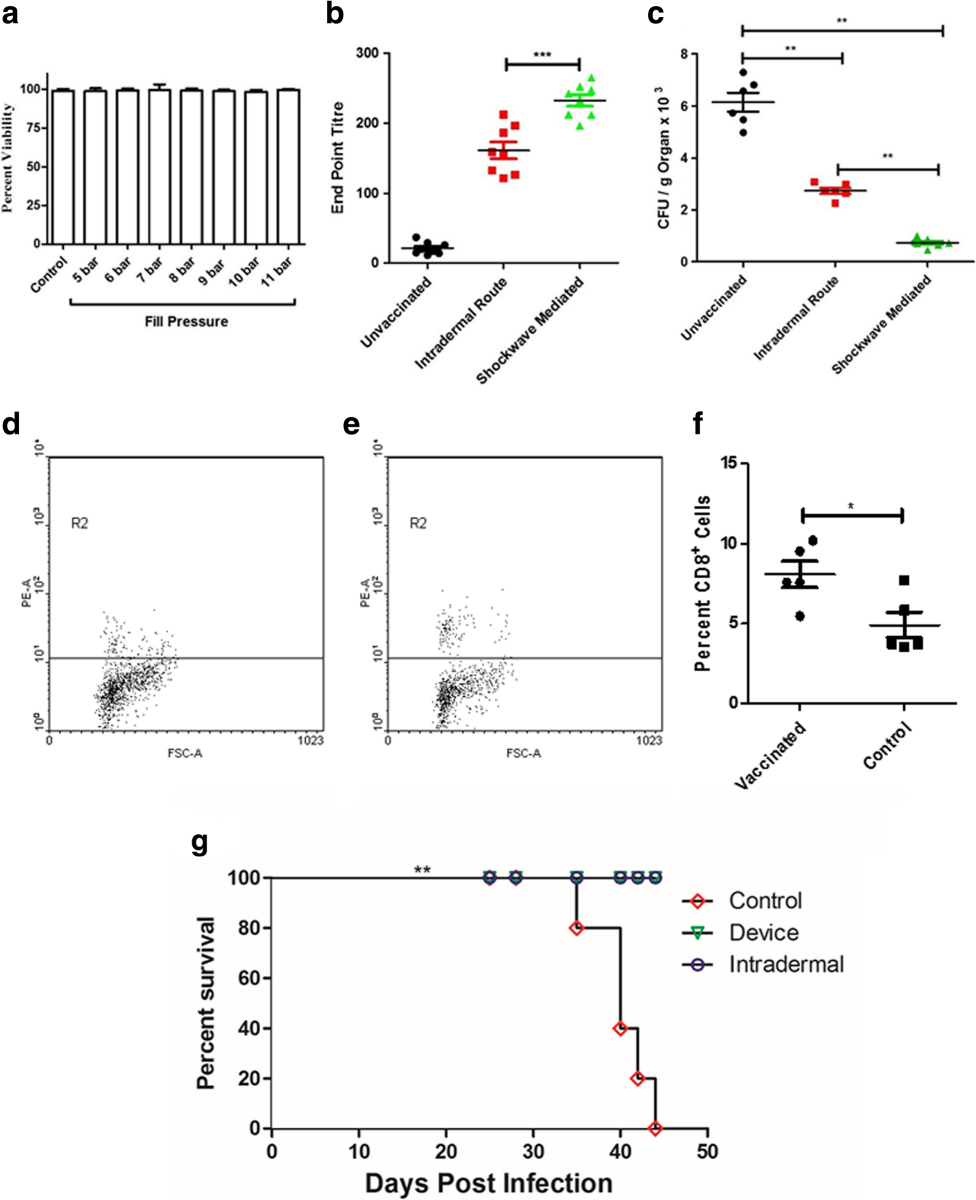
Efficiency of BCG vaccine delivery using the device. **a** *M.bovis* BCG stationary phase cultures were exposed to shockwaves at different pressures to check the viability of bacteria; unexposed cultures served as the control group (**b**) Primary and a booster dose was delivered using device, orally and through intradermal route to mice. The serum IgG levels of BCG specific IgG were tested using ELISA; PBS delivered using the device was used as control; bar shows the mean value of the experiments. Error bar shows standard deviation (s.d.). (*P* value - Student’s *t*-test). (**c**) BCG was administered to mice using the device and via intranasal route. A booster dose was administered at 1-week post-immunization. 7 days’ post-booster dose, mice were infected via intranasal route with a lethal dose (10^8^ CFU/mouse) of *Mycobacterium tuberculosis* H37Ra strain*.* 14 days after challenge, lungs were aseptically isolated and dissected to enumerate the Mycobacterial burden. Statistical significance is specified as **P < 0.005 (Two-way ANOVA test). **d**–**f** Splenic T cells were isolated from unvaccinated and vaccinated mice. Cells were stained with PE-tagged antiCD8 antibody (MiltenyiBiotec) and were analyzed by flow cytometry. Numbers of CD8+ cells were compared between the unvaccinated and the vaccinated cohort. **g** Mice (*n* = 5 per group) were infected with lethal dose (10^8^ CFU/mouse) of virulent *Mycobacterium tuberculosis*,7 days after immunization as described in the previous experiment, and the survival of mice was estimated till day 45 post challenge. [*P* < 0.0001 (Log rank test)]

BCG vaccine is conventionally administered in the intradermal region. Using shock waves to deliver this vaccine was more clinically relevant. We began with checking the effect of shockwaves on the viability of the vaccine strain. It was observed that shock waves had no effect on the viability of the vaccine strain (Fig. 4a). Animals were vaccinated using the device in the intradermal region of the abdomen. A booster dose was also administered to enhance the immune protection. The antibody titer against BCG was found significantly higher in the cohort vaccinated using the device as compared to the conventionally vaccinated route (Fig. 4b). This highlights the enhanced immune protection conferred when vaccinated using the device. To validate the immune response further, the vaccinated animals were challenged with *M. tuberculosis H37Ra* via the nasal route. The bacterial burden in the lungs of the animals vaccinated using the device was significantly lower than that of the conventionally vaccinated group as well as the control group (Fig. 4c). The CD8+ cells were estimated in from the spleen. There was a significant increase in the cytotoxic T cells in the vaccinated cohort in comparison to the control group (Fig. 4d-f). To perform a stringent test of vaccination, mice vaccinated with BCG using the device as well as the conventional intradermal route were infected via the nasal route with a lethal dose of virulent *Mycobacterium tuberculosis* H37Rv and were monitored for mortality. A group of animals were injected PBS as a mock vaccination. It was observed that the animals vaccinated using the device and the ones vaccinated via the intradermal route showed 100% survival as compared to the mock vaccinated group. The entire control group succumbed to infection by day 44 post challenge **(**Fig. 4g **)**. All these results prove that vaccination done using shockwave device is highly efficient in all the model organisms and infections tested.

### Efficiency of salmonella vaccine delivery using shockwave device

**Fig. 5:**
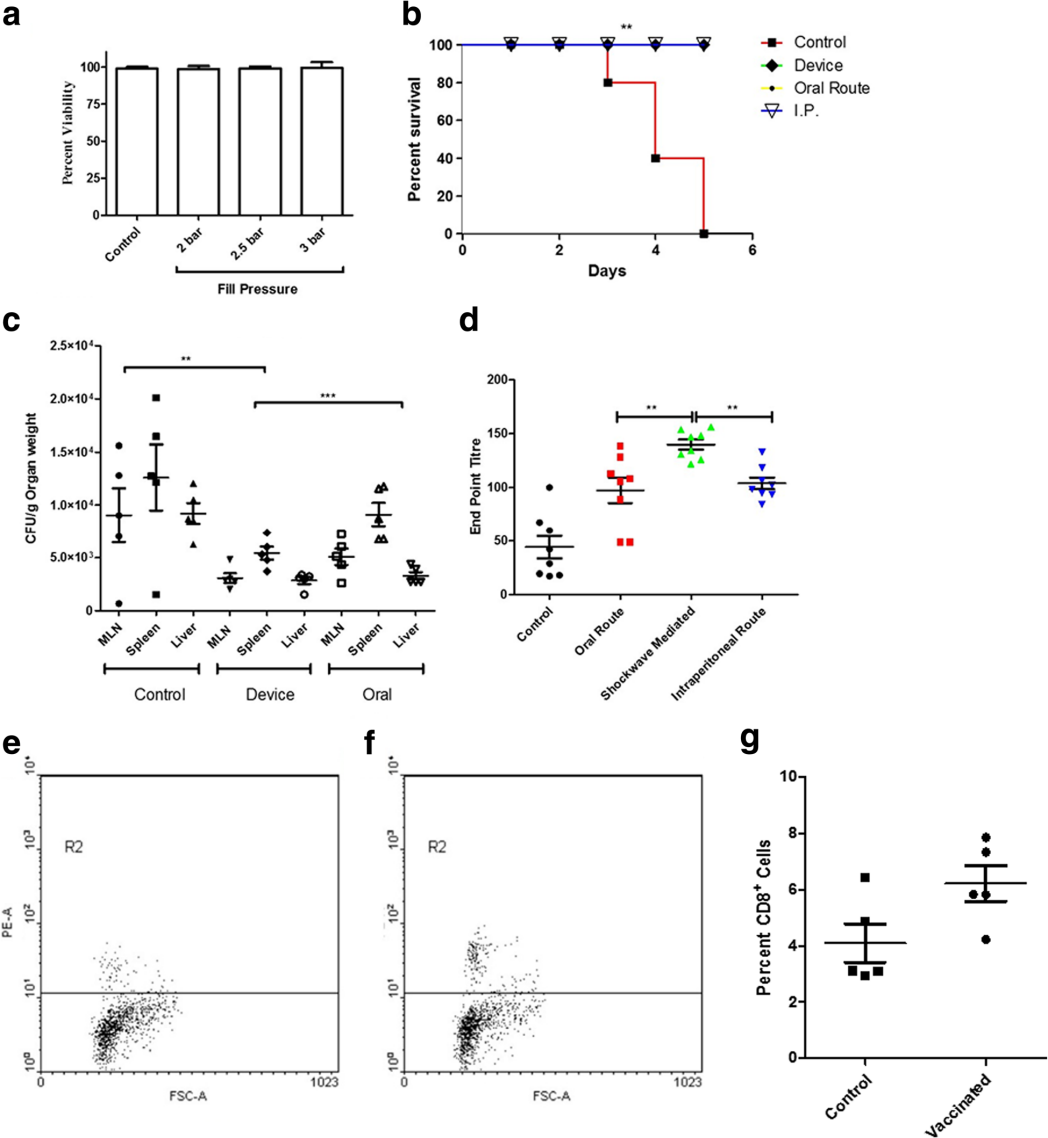
Efficiency of Salmonella vaccine delivery using the device. **a** *Salmonella* Typhimurium stationary phase cultures were exposed to shockwaves at different pressures to check the viability of bacteria; unexposed cultures served as the control group (**b**) Mice (*n* = 5 per group) were infected with lethal dose (10^8^ CFU/mouse) of virulent *Salmonella* orally, 5 days after immunization as described in the previous experiment, and the survival of mice was estimated. [*P* < 0.0001 (Log rank test)]. **c** Single dose of DV-STM-07 was delivered using device, orally and through I.P route and the serum IgG levels were tested against *Salmonella*-specific Lipopolysaccharide (LPS) using ELISA; PBS delivered using the device was used as control; bar shows the mean value of the experiments. Error bar shows standard deviation (s.d.). (*P* value - Student’s *t*-test). **d** DV-STM-07 was administered to mice using the device and via oral route. Phosphate buffered saline (PBS) delivered using the device was used as control. Mice were infected with a lethal dose (10^7^ CFU/mouse) of virulent strain of *Salmonella* at 1-week post-immunization*.* 5 days after the oral challenge, MLN, spleen and liver were aseptically dissected for checking the *Salmonella* burden. Statistical significance is specified as **P < 0.005 (Two-way ANOVA test). **e**–**g** Splenic T cells were isolated from unvaccinated and vaccinated mice. Cells were stained with PE-tagged antiCD8 antibody (Miltenyi Biotec) and were analyzed by flow cytometry. Numbers of CD8+ cells were compared between the unvaccinated and the vaccinated cohort

Shockwaves had no significant effect on the viability of the vaccine strain DV-STM-07 (Fig. 5a). The survival rate of mice immunized using the device was similar to the group immunized via the oral and the IP route, whereas the mice from the control group did not survive when challenged with virulent *Salmonella* (Fig. 5b)**.** This clearly proves the improved efficiency of the vaccination performed using the device. The bacterial colonization in liver, spleen and mesenteric lymph nodes of mice vaccinated using the device was significantly lower than that in the control, as well as, in the mice immunized by the oral and IP route (Fig. 5c). The level of serum IgG in the mice vaccinated using the shockwave devices were significantly higher than in the other modes of vaccination (Fig. 5d). The number of CD8+ cells in the cohort of animals vaccinated using the device was significantly high as compared to the animals vaccinated via the conventional oral route (Fig. 5e–g).

### Molecular mechanism of enhanced immune response to vaccine administered using shockwave device

**Fig. 6:**
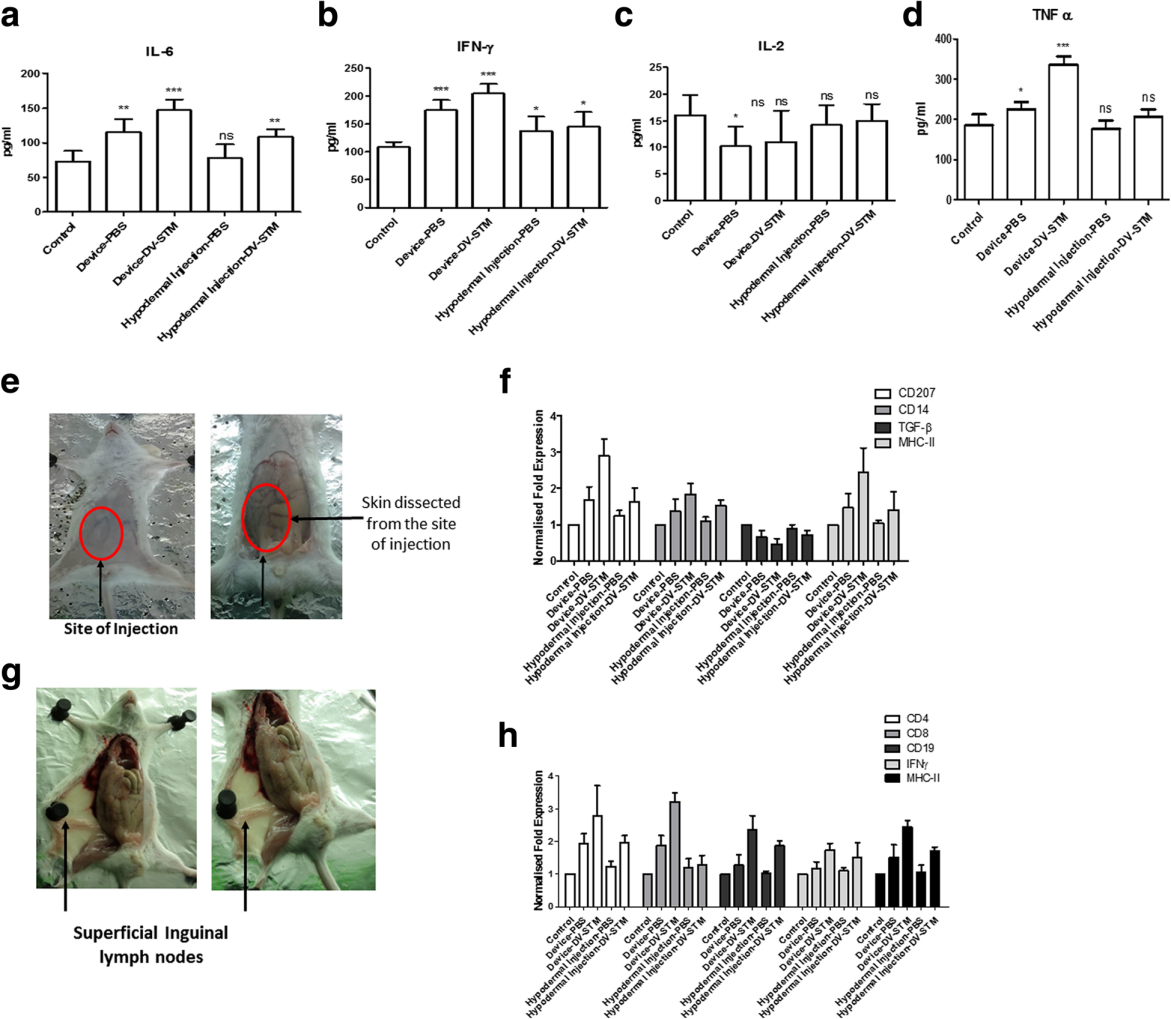
Molecular mechanism of immune response to shockwave mediated vaccine delivery. **a**–**d** Cytokine levels namely IL-8, IFN-γ, TNF-α, IL-2, measured by ELISA post vaccination in the serum. **e**, **g** Actual photographs showing the isolation of skin from the site of injection and isolation of superficial lymph nodes for RNA isolation and qRT PCR analysis of various markers for activation of immune response. **f** Normalized fold expression of various genes CD207, CD14, TGF-ß and MHC-II from the site of injection. **h** Normalized fold expression of genes CD4, CD8, CD19, MHC-II and IFN-γ from the lymph nodes of vaccinated and control animals. The markers are seen to be upregulated in the vaccine delivered using the device

The immune response generated by the shockwave mediated vaccine delivery was found to be significantly higher than the conventional intradermal injections of the same vaccine. This intrigued us to further study the effect of shockwave in priming the immune system. In order to study the effect mentioned above, we designed the experiment where a cohort of animals was administered PBS using the shockwave device. A similar cohort was administered PBS using a hypodermal needle syringe. Post vaccination cytokine levels were checked in the animals. Cytokines namely, IL-8, IFN-γ, TNF-α and IL-2 were compared. These cytokines are important in generating a potent immune response by activating the immune cells. The levels of these cytokines were found to be high in the vaccine delivered using the device as compared to vaccine delivered using hypodermic syringe **(**Fig. 6a–d **)**. Comparison of the above two cohorts was also done at the molecular level where the expression of genes like CD207, CD14, TGF-ß, MHC-II from the site of injection 24 h post injection **(**Fig. 6e **)** and CD4, CD8. CD19, IFN γ and MHC-II were evaluated from the superficial inguinal lymph nodes day 3 post injection using qRT-PCR **(**Fig. 6g **)**. These factors mainly reflect the status of immune response generated against an antigen introduced through the dermal route. CD207 is the exclusive marker for skin resident dendritic cells called as the Langerhans’s cells [34]. These cells are the most potent antigen presentation machinery present in the skin. MHC-II is the major histocompatibility complex associated with exogenous antigen processing and presentation [34]. CD4 and CD8 are the markers for helper T cells and cytotoxic T cells respectively. CD19 is the surface marker for antibody producing plasma cells [35]. It was observed that the markers tested at the site of injection were up- regulated at 24 h post injection in the PBS injection using device as compared to PBS injection using hypodermic syringe **(**Fig. 6f **)**. Cohort injected with the vaccine strain showed significant up-regulation of the markers. Day 4 post injection, molecular markers in the lymph nodes was quantitated. CD4, CD8, MHC-II, CD19 and IFN-γ all were found up-regulated in the PBS injection using device cohort. The up-regulation was significant in the cohort injected with vaccine strain using the device **(**Fig. 6h **)**. This up-regulation of the immune markers even in the case of PBS injection using the device clearly demonstrates that the shockwave and the high velocity jet which hits the site of injection activate the immune system. This might be because of the fact that shockwaves are recognised as a condition of physical stress and various stress responses are rapidly mounted against shockwaves used for vaccine delivery. Therefore, all these results highlight the role of shockwaves in eliciting an immune response independent of the vaccine being administered.

### Mechanism of enhanced vaccine delivery using the present device

The shockwave produced by the detonation of the oxyhydrogen mixture is the driving force for needleless vaccination. A large portion of the incident shockwave energy passes through the silicone rubber and is felt by the vaccine placed in the cavity. While some part of the incident energy is absorbed by the membrane itself another portion of the energy is reflected back into the shock tube. Additional file 1: Figure S1 shows the pressure experienced by the vaccine placed in the cavity during operation of the device. The incident shockwave in the driven section of the shock tube has a peak pressure about 16 bar (See Fig. 2a). But it gets amplified to about 200 bar in the liquid as seen in Additional file 1: Figure S2. The pulse duration lasts for about 40 microseconds. The rise in pressure inside the cavity to a pressure of up to 200 bar could be due to the combined effect of the transmitted shockwave and the pressure rise in the liquid due to the bulging of the rubber membrane. The gradual pressure drop after the peak value is due to rubber going back to its original shape and thus creating a pressure relieving effect in the cavity. There are some high frequency oscillations can be observed in the fall time duration which slowly dampen out with time. This could be because of the vibration of the silicone membrane or the multiple reflections in the cavity. The time period of these oscillations corresponds to the multiple reflections of pressure waves in the cavity rather than the natural frequency of vibration of silicone rubber (see Additional file 1: Note S1 and Note S2). Therefore, the transmitted shock wave undergoes a series of reflections against the bottom of the cavity and the face of the rubber membrane causing high frequency oscillations in the pressure inside the cavity and also continuously attenuating in intensity. Keeping all these points in mind, illustrations of the possible mechanism of vaccination using the shockwave assisted device have been shown in Additional file 1: Figure S3.

## Discussion

Shockwave-assisted vaccine delivery surpasses the present-day needle and syringe technique of injections as it is needle-free, painless, eliminates the need for non-biodegradable waste disposal, avoiding the risk of disease transmission and there is no damage to cell tissues around the region of injection. In addition to these advantages, the proposed device uses in situ generation of oxyhydrogen mixture which avoids storage and mixing of detonable gases. The device uses minimal consumables, produces water as a main by-product during usage and is safe as well as reproducible. All these advantages coupled with the innovative design make it a unique and first-of-its-kind device. The device was optimized to deliver vaccines in the transdermal region which lies at a depth of ~100 μm from the skin surface. Characterization of the shockwave generated was done using the conventional Schlieren imaging technique. The velocity of the fluid jet formed, was measured using a high-speed camera and was found out to be 94 ms^−1^. Theoretically calculated values of velocity of the liquid jet gives similar results (see Additional file 1: Note S3 and Figure S2). The depth of deposition of the fluid using the shockwave device was measured in both artificial skin model of polyacrylamide and in the actual murine skin by injecting fluorescently-labeled beads. The depths of fluid delivery were found out to be 120 μm and 80 μm in the artificial skin and murine skin respectively. These depths are also indicative of the absence of free nerve endings thus making the delivery system pain-free. The depth of penetration of the liquid is directly proportional to the kinetic energy of the fluid jet which in turn is governed by the strength of the shockwave driving the formation of the jet. The strength of the shockwave can be tuned so as to achieve the desired depth of penetration of the liquid jet. Further, vaccine delivery was tested in the murine model for two well established bacterial vaccines namely, DV-STM-07 and the BCG vaccine. Suitable animal models were chosen for the experiments. Vaccinations performed by using the device were scored at all possible levels of a virulent challenge by *Salmonella* Typhimurium and *Mycobacterium tuberculosis* H37Ra. The survival of the vaccinated animals was at par with the cohort vaccinated by the conventional methods. Immune response is measured by the production of pathogen-specific immunoglobulins in the host. High level of antibody production indicates the robustness of vaccination. The IgG level in the shockwave mediated vaccine delivery was found out to be significantly higher as compared to the vaccination by the conventional route in both DV-STM-07 and BCG vaccine strains. Next, the efficiency of the vaccination lowering the pathogen burden in the systemic organs was evaluated. It was found that the animals vaccinated using the device had lower level of pathogen colonization in liver, spleen and mesenteric lymph nodes for *Salmonella* and in the lungs for *M.tuberculosis* H37Ra which wasstatistically significant as well. Production of cytotoxic T cells (CD8^+^ cells) also indicates an elicited immune response. These cells in the splenic cell population were enumerated in the vaccinated and the control cohort of animals. The vaccinated cohort showed a statistically significant higher number of these cells as compared to the control group for both the vaccine strains. The vaccination efficiency was also evaluated by challenging the vaccinated animals with virulent *Mycobacterium tuberculosis*. It was observed that the vaccinated animals show a 100% survival as compared to the mock vaccinated cohort. Insights into the molecular mechanism of immune response to vaccine delivered using the device revealed the role of shockwaves alone in priming the immune response. Cytokine profile in the animals vaccinated using shockwave device show a significant increase as compared to control and hypodermal syringe injections. The molecular markers like CD207, CD19, CD4, CD8 etc. indicative of the status of immune system showed significant up-regulation in the animals vaccinated using the shockwave device. These observations form firm evidence that the shockwave device developed in this study bears the potential to deliver a range of vaccines and drugs to the desired locations by altering the operating conditions. This device has a promising future in assisting clinicians and healthcare professionals to perform large scale needle-less and pain-free vaccine/drug delivery. The recent advances in photo electrolysis of water and development of new photoelectrode materials can aid in further miniaturization and increased efficiency of the device.

## Conclusion

A novel device was developed and optimized for intra dermal vaccine delivery in murine model. Conventional as well in-house developed vaccine strains were used to test the system. It was found that the vaccine delivery and immune response was at par with the conventional routes of vaccination. Thus, the device reported can be used for delivering live attenuated vaccines in the future.

## Additional file

Additional file 1: Supplementary Dataset.
**Figure S1.** Working principle of the oxyhydrogen detonation-driven miniature shock tube. **Figure S2.** A plot showing the velocity of the jet calculated theoretically from the pressure measured inside the cavity. **Figure S3.** Proposed mechanism enhanced vaccine delivery using the device. **NOTE S1.** Estimation of natural frequency of silicone rubber clamped at the edges. **NOTE S2.** Estimation of time taken by stress waves to travel along liquid column. **NOTE S3.** Theoretical estimation of velocity of liquid jet. (DOCX 438 kb)
Additional file 2: video 1. Schlieren video of the blast evolution from the open end of the device. The initial oxyhydrogen fill pressure is 2.5 bars. (XSPF 564 bytes)
Additional file 3: video 2. Schlieren video of the liquid jet containing the drug. The initial oxyhydrogen fill pressure of 2.5 bars. (XSPF 564 bytes)

## Abbreviations

BCG: Bacillus Calmette–Guérin
CD: Cluster of differentiation
CFU: Colony forming unit
IgG: Immunoglobulin G
IP: Intraperitoneal
LPS: Lipopolysaccharides
PBS: Phosphate buffered saline
PCR: Polymerase chain reaction
qRT-PCR: Quantitative real time polymerase chain reaction
RBC: Red blood cell
