# Supplementary material for: Insights into the mechanism of a novel shockwave-assisted needle-free drug delivery device driven by in situ-generated oxyhydrogen mixture which provides efficient protection against mycobacterial infections
Source: J Biol Eng. 2017 Dec 12;11:48. doi: 10.1186/s13036-017-0088-x (PMC5727940; doi:10.1186/s13036-017-0088-x)
Supplement: Additional file 1: Supplementary Dataset. — Figure S1. Working principle of the oxyhydrogen detonation-driven miniature shock tube. Figure S2. A plot showing the velocity of the jet calculated theoretically from the pressure measured inside the cavity. Figure S3. Proposed mechanism enhanced vaccine delivery using the device. NOTE S1. Estimation of natural frequency of silicone rubber clamped at the edges. NOTE S2. Estimation of time taken by stress waves to travel along liquid column. NOTE S3. Theoretical estimation of velocity of liquid jet. (DOCX 438 kb) [file 13036_2017_88_MOESM1_ESM.docx]

**SUPPLEMENTARY FIGURES**


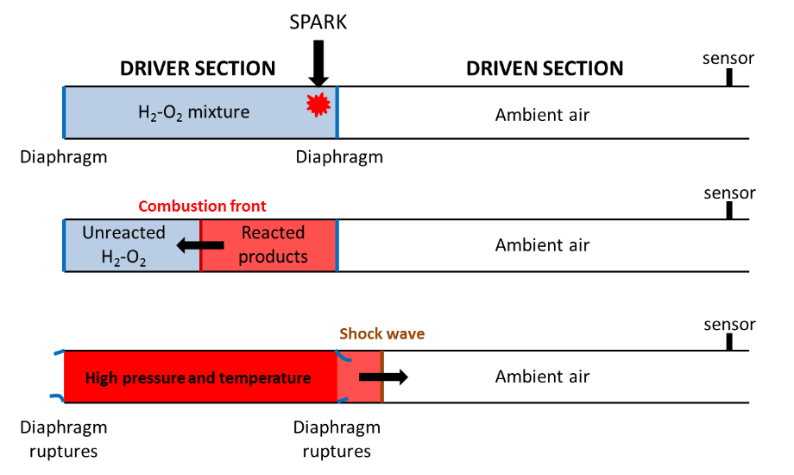


**(b)**

**(a)**


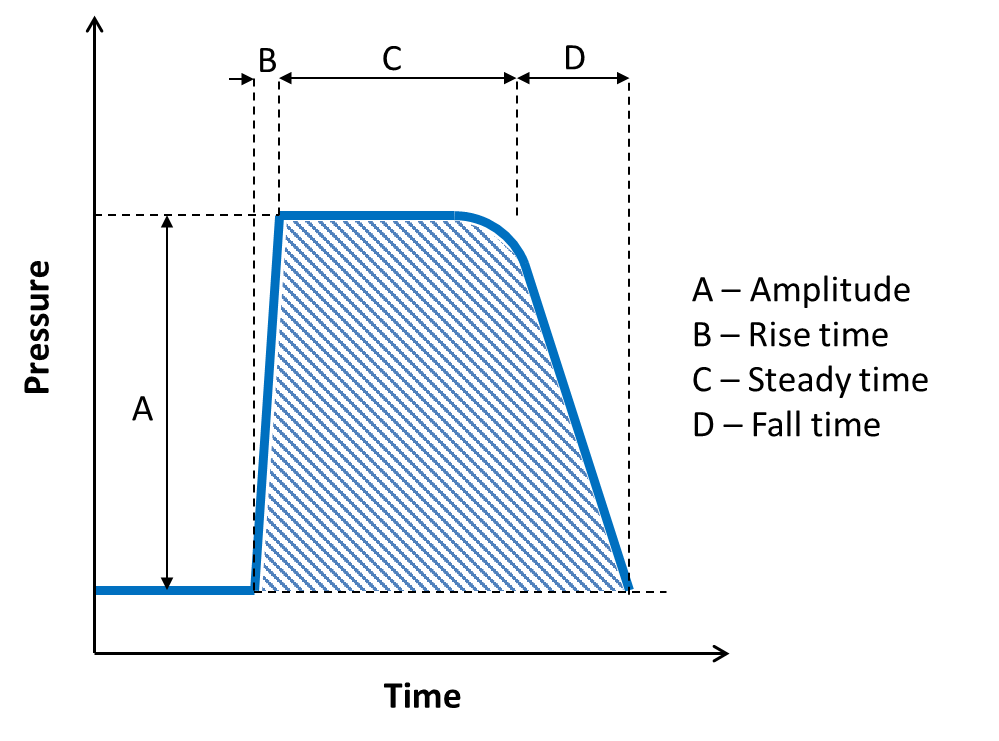


**Figure-S1. Working principle of the oxyhydrogen detonation-driven miniature shock tube:** **(a)** A pictorial representation of the different stages of working of an oxyhydrogen combustion driven shock tube. **(b)** The typical pressure signal of a shock wave generated as measured by a pressure sensor located at the end of the driven section [indicated in (a)]. The shaded region represents the impulse generated by the shock wave.


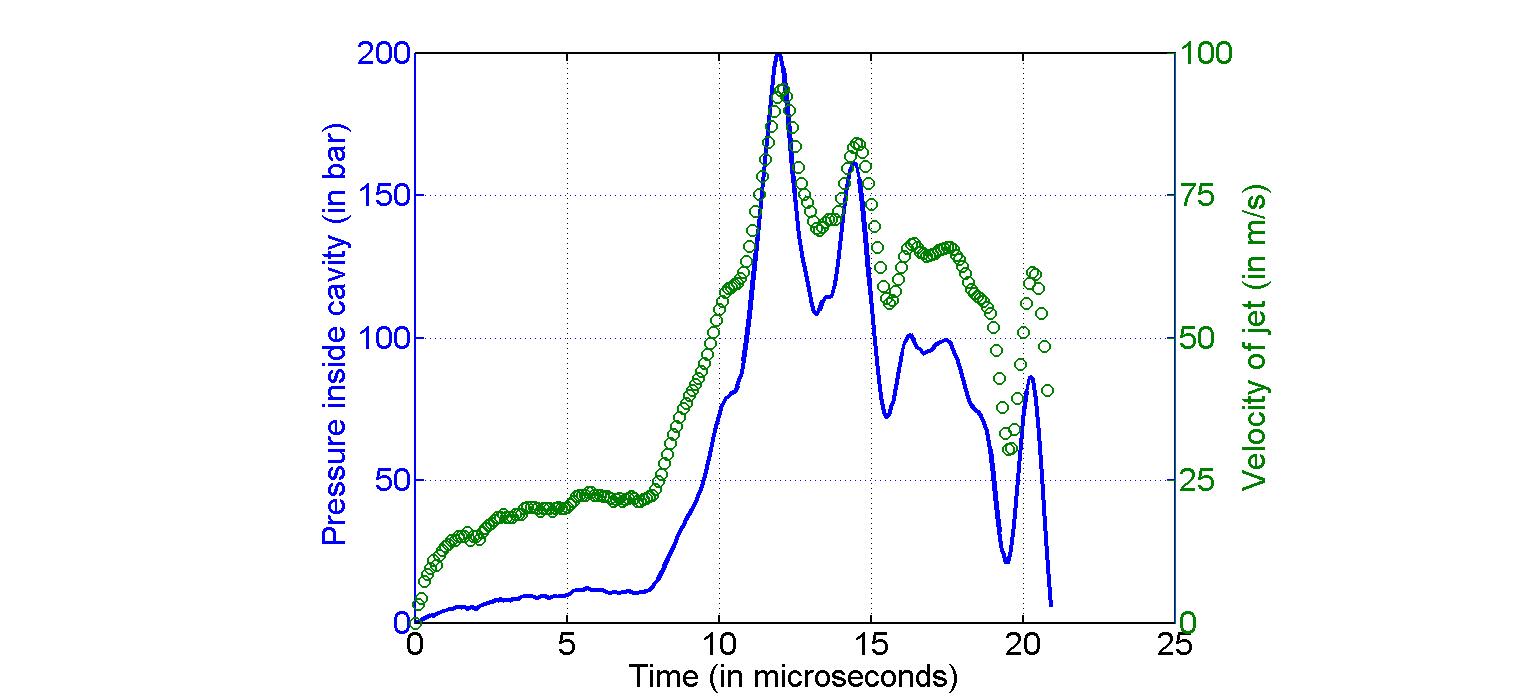


**Figure-S2. A plot showing the velocity of the jet calculated theoretically from the pressure measured inside the cavity.**


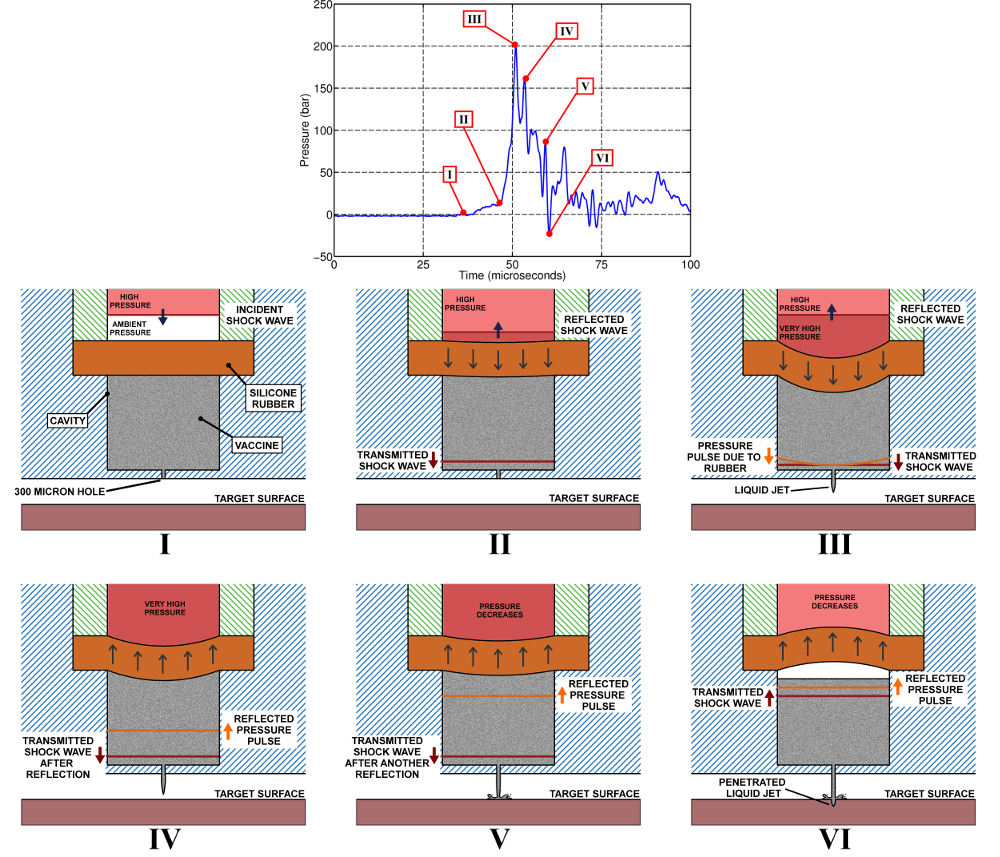


Figure 6: Proposed mechanism of liquid jet delivery using shockwaves

**Figure S3: Proposed mechanism enhanced vaccine delivery using the device**

**SUPPLEMENTARY NOTES**

NOTE S1: Estimation of natural frequency of silicone rubber clamped at the edges

The angular natural frequency ($\omega_{n}$) for a circular plate clamped at the edges is given by^1^,

**Equation 1:**

$$\omega_{n}(in rad/s)=B\sqrt{\frac{Et^{3}}{\rho a^{4}\left( 1-\vartheta^{2} \right)}}$$

where,

$E$ – Young’s modulus (Pa)

$t$ – Thickness of plate (m)

$\rho$ – Density of material (kg/m^3^)

$a$− Diameter of plate (m)

$\vartheta$ – Poisson’s ratio

The value of *B* for the first mode of vibration is 11.84. The thickness of the silicone rubber used for the present work is 2mm while the exposed diameter of the silicone rubber is 6mm which is same as the inner diameter of the shock tube. Since, the exact values of the mechanical properties of the silicone rubber used is not known, a range of values for the mechanical properties of silicone rubber is used for calculations and is tabulated below^2^.

|  | **Maximum** | **Minimum** |
| --- | --- | --- |
| $\boldsymbol{E}$ | 50 MPa | 1 MPa |
| $\boldsymbol{\rho}$ | 2300 kg/m^3^ | 1100 kg/m^3^ |
| $\boldsymbol{\vartheta}$ | 0.49 | 0.47 |

Substituting the corresponding values in equation 1, the value of ‘$\omega_{n}$’ for the maximum and minimum values of mechanical properties is found to be 4975 rad/s and 1005 rad/s respectively. The natural frequency $\left( \nu=\frac{\omega_{n}}{2\pi} \right)$ corresponding to these values are 791 Hz and 160 Hz respectively. Therefore, the time period ($t=\frac{1}{\nu}$) corresponding to the natural frequency is 1263 *µ*s and 6250 *µ*s respectively.

**References:**

^1^ Harris CM, Piersol AG. *Harris' shock and vibration handbook. Sixth Edition.* New York: McGraw-Hill; (2010).

^2^ http://www.azom.com/properties.aspx?ArticleID=920

NOTE S2: Estimation of time taken by stress waves to travel along liquid column

The speed of sound in a medium is given by,

$$c=\sqrt{\frac{K}{\rho}}$$

$K$ – Bulk modulus of the medium

$\rho$ – Density of the medium (considered close to density of water)

Hence, the speed of sound in water is approximately 1500 m/s. Therefore, time taken by acoustic wave to travel in a liquid column of height 5mm is 3.33*µ*s.

NOTE S3: Theoretical estimation of velocity of liquid jet

The typical pressure signal measured in the cavity has already been shown in the **Fig. 6**. Using this pressure plot, the mass flow rate ‘$Q$’ can be estimated as a function of time. The mass flow rate ‘*Q*’ of an incompressible liquid from an orifice is given by,

$$Q(t)=A_{2}\sqrt{\frac{2(P_{1}(t)-P_{2})\rho}{1-{(A_{2}/A_{1})}^{2}}}$$

where ‘$A_{1}$’ is the area in the cavity, ‘$P_{1}(t)$’ is pressure in the cavity, ‘$A_{2}$’ is area of orifice, ‘$\rho$’ is density of the liquid and ‘$P_{2}$’ is ambient pressure.

For a small time ‘$dt$’, the mass ‘$dm$’ ejected from the cavity can be calculated. The change in momentum of the ejected mass ‘$dm$’ gives the impulse ‘$J$’ provided to the mass.

$$J= dm \times(v_{2}-v_{1})$$

where ‘$v_{1}$’ is the initial velocity (= 0) and ‘$v_{2}$’ is the final velocity attained.

The impulse can be calculated by finding the area under the pressure-time plot for the time duration ‘$dt$’ and multiplying it by the area of orifice ‘$A_{2}$’. Substituting for the impulse in the above equation, the velocity of the ejected mass of liquid can be obtained as a function of time.

The pressure inside the cavity as a function of time that is considered for the calculation is shown in blue in the **Supplementary Fig. S2**. Only the first positive curve of the pressure-time plot is considered. A simple program has been written to obtained the velocity as a function of time. The velocity of the jet obtained as a function of time is shown in green in the **Supplementary Fig. S2**. The maximum value obtained for the velocity of the jet is 93.5 ms^-1^.

**Supplementary Video 1:** Schlieren video of the blast evolution from the open end of the device. The initial oxyhydrogen fill pressure is 2.5 bars.

**Supplementary Video 2:** Schlieren video of the liquid jet containing the drug. The initial oxyhydrogen fill pressure of 2.5 bars.
